# Survey of Computational Algorithms for MicroRNA Target Prediction

**DOI:** 10.2174/138920209789208219

**Published:** 2009-11

**Authors:** Dong Yue, Hui Liu, Yufei Huang

**Affiliations:** 1 Department of Electrical and Computer Engineering, University of Texas at San Antonio (UTSA), San Antonio, TX 78249-0669, USA; 2 SIEE, China University of Mining and Technology, Xuzhou, Jiangsu 221008, China; 3 Greehey, Children's Cancer Institute and Department of Epidemiology and Biostatistics at the University of Texas, Health Science Center at San Antonio (UTHSCSA), San Antonio, TX 78229, USA

## Abstract

MicroRNAs (miRNAs) are 19 to 25 nucleotides non-coding RNAs known to possess important post-transcriptional regulatory functions. Identifying targeting genes that miRNAs regulate are important for understanding their specific biological functions. Usually, miRNAs down-regulate target genes through binding to the complementary sites in the 3' untranslated region (UTR) of the targets. In part, due to the large number of miRNAs and potential targets, an experimental based prediction design would be extremely laborious and economically unfavorable. However, since the bindings of the animal miRNAs are not a perfect one-to-one match with the complementary sites of their targets, it is difficult to predict targets of animal miRNAs by accessing their alignment to the 3' UTRs of potential targets. Consequently, sophisticated computational approaches for miRNA target prediction are being considered as essential methods in miRNA research.

We surveyed most of the current computational miRNA target prediction algorithms in this paper. Particularly, we provided a mathematical definition and formulated the problem of target prediction under the framework of statistical classification. Moreover, we summarized the features of miRNA-target pairs in target prediction approaches and discussed these approaches according to two categories, which are the rule-based and the data-driven approaches. The rule-based approach derives the classifier mainly on biological prior knowledge and important observations from biological experiments, whereas the data driven approach builds statistic models using the training data and makes predictions based on the models. Finally, we tested a few different algorithms on a set of experimentally validated true miRNA-target pairs [1] and a set of false miRNA-target pairs, derived from miRNA overexpression experiment [2]. Receiver Operating Characteristic (ROC) curves were drawn to show the performances of these algorithms.

## 1. INTRODUCTION

In classical molecular biology, the functional units in a genome are genes or the DNA regions that code proteins. The non-coding regions were considered as nonfunctional, or junk DNAs. However, the notion has been seriously challenged ever since the discovery of RNA interference (RNAi), a technology considered as one of the most exiting breakthrough in biology in the past decade and was accordingly awarded 2006's Nobel Prize in Physiology. Since then, many types of non-coding RNAs have been identified as important regulatory elements in mammalian and non-mammalian cells, and microRNAs (miRNAs) have drawn increasing research attention among these non-coding RNAs. MicroRNAs are a class of single-stranded non-coding RNAs with about 19 to 25 nucleotides (nts) in length, which are mostly known to inhibit the translation of mRNAs into proteins or promote repression of mRNA expression [3, 4]. In human, more than 500 miRNAs have been annotated in the miRNA registry (MirBase) [5, 6] with over 1000 miRNAs predicted to exist. These miRNAs are believed to directly regulate around 30% of human protein coding genes and each miRNA would mediate the expression of on average over 200 genes. Given these facts, miRNAs inevitably play important regulatory roles in many biological processes and diseases including cell development [7, 8], stress responses [9, 10], viral infection [11, 12], and cancer [13-15]. For example, human miR-155 has been shown to regulate T helper cell differentiation and mediate the T cell-dependent antibody response [16, 17] and it has also been implicated in a number of cancers including Burkitt's and Hodgkin lymphomas, breast cancer, lung, and colon cancers [18-20]. Also, the miRNA cluster miR-17-92 is indicated to be a potential oncogene enhancing cell proliferation [21, 22] and has been associated with several types of cancer including colorectal cancer [23] and lung cancer [24]. Three studies [22, 25, 26] have also established that the specific miRNAs are expressed in most common cancers and demonstrated the effects of miRNAs on cancer development. miRNAs have also been used for the diagnosis, prognosis and response to treatment of cancer patients. It is foreseen that their role will be extended in the future to therapeutic approaches, in particular to identify new therapeutic targets. As a result, miRNA research has been very active and named as one of the areas to watch and make breakthrough of the year 2007 by the Science magazine [27].

Despite their importance, the *in vivo* functions of most human miRNAs are still poorly understood. The reality is manifested by the fact that only about 1000 human miRNA target genes have been experimentally validated, a faction of the potentially human gene targets. As a result, the global pattern of cellular functions and pathways that are affected by miRNAs in various diseases remains largely unknown. Understanding the biological functions of miRNA is therefore one of the main goals of current miRNA study and identifying regulatory targets of miRNAs is the critical first step. In part due to the sheer number of miRNAs and their potential targets, a mere experiment based prediction design is extremely laborious and economically unfavorable. Alternatively, computational target prediction methods coupled with high-throughput experiments can provide valuable clues for potential targets and more efficiently generate manageable hypotheses for experiments.

Given the importance of the topic, we provided a timely survey of the computational algorithms for miRNA target prediction in this paper. Computational target prediction algorithms came to exist since TargetScan [28-30] was proposed in 2003, which is a rule-based algorithm and still among the most popular algorithms nowadays. Restricted mainly by the availability of relevant data, early target prediction algorithms are largely rule-based, in which the target is predicted based on simple discriminative rules derived from important features of target recognition observed from experiments. The rule-based algorithms include TargetScan [28-30], miRanda [31-33], PITA [34], etc. In recent years, new data-driven prediction algorithms emerge along with the improving knowledge of miRNA target recognition and the increasing availability of various types of relevant data sets. Data driven algorithms rely on important discriminative features learned from data using sophisticated models. The data driven algorithms include MirTarget [35, 36], PicTar [37], miTarget [38], etc. We discussed the computational details of these algorithms and summarized relevant data sources in this paper. We are aware that there exists good surveys on miRNA target prediction including articles [39-44], each addressing the survey from a different perspective. Although their coverage and depth are adequate for the intended audience, they nevertheless lack the discussions of issues closer to the computation community. First, the majority focus the survey only on the rule-based algorithms and are short of addressing important advances in data driven algorithms, which also utilize other data types. Secondly, most of them provide little implication on the connections and difference among the different algorithms and they rarely concern the performance of these algorithms. As a result, it is difficult for readers to perceive the pros and cons of different algorithms. In light of the importance of the topic, the goal of this survey is to emphasize the computations and models of each algorithm and try to provide insights into the advantage and disadvantages of these computational miRNA target prediction.

The rest of paper is organized as follows. In section 2, the background of miRNA target recognition is provided and relevant data resources for target prediction are included. In section 3, the general problem of computational target prediction is formulated mathematically and important features for target prediction are enlisted and discussed. Then, the rule-based algorithms are surveyed in details followed by the thorough discussion of various data-driven algorithms. In section 4, the validation result of a few algorithms based on experimental validated targets is presented. Conclusion is drawn in section 5.

## 2. PRINCIPLES OF miRNA TARGET RECOGNITION AND PREDICTION ONLINE SOURCE

An important initial step of analyzing miRNA to perform the regulatory task is to recognize its target genes. Although the detailed target recognition mechanism is still elusive, the consensus suggests that the Watson base pairing of miRNA with its targets' mRNAs is the key. In performing the base paring, the mature miRNA is first assembled into the effector protein complexes called miRNPs, which share many similarities to the RNA-induced silencing complex (RISC). It is also clear that all miRNAs are bound to a minimum effector complex that contains an Argonaute (Ago) protein. Once the miRNP is assembled, the miRNA guides the complex to its targets by the base-pairing with targets' mRNA. Base paring mostly occurs at the 3' untranslated region (UTR) of a target gene, although the paring is observed in a few cases to exist also in the 5' UTR and coding regions. The most elusive fact of target recognition is that the base-paring within the target mRNA is almost always imperfect. Regulatory effect has been observed for the pairing of as little as 8 base-pairs between miRNA and its target mRNA [45]. The lack of specificity in perfect base pairing creates enormous difficulty to understand the mechanism in target recognition. Existing research suggests a few distinct features about miRNA base paring. Particularly, the perfect pairing has been noted with much higher frequency in the so-called “seed” region, often defined as the 2nd-8th nt from the 5' end of the miRNA [30]. Experiments indicate the G-U wobble pairs and bulges in the seed region significantly interrupt the miRNA-target interaction [45]. However, perfect pairing is neither necessary nor sufficient for miRNA-target interaction as let-7 [46] in C. *elegans*. Yet, the non-ideal pairing in seed region can be compensated by the additional complimentary at the 3' end of the miRNA as miR-24 [47] in Homo sapiens. Furthermore the sequence context outside of the binding site regions has also been shown to impact binding as miR-199b [47] in Homo sapiens. Using these sophisticated yet flexible target recognition schemes, a miRNA is estimated to target on average hundreds of mRNAs. In addition, the 3' UTR of the target mRNA can contain multiple sites and the presence of multiple sites tends to increase the possibility of binding [30, 45].

### Inhibition of Translation or Repression of mRNA Expression

miRNA is mostly known to down-regulate target mRNAs, although few recent works emerge to show its potential up-regulative role. Increasing evidence indicates that the miRNA controls two regulatory modes which includes inhibition of translation and repression of mRNA expression. The latter one can be also accomplished by three different mechanisms ranging from mRNA degradation to mRNA deadenylation to mRNA sequestration. The precise factors to determine regulatory mode are still poorly understood. Many recent evidence suggest that translational repression can be considered as the primary event and any reduction of mRNA levels is a possible secondary effect of translational repression. In many cases, mRNA degradation cannot be accounted for the translational repression.

### Data Resource for miRNA Target Prediction

To predict targets computationally, various data including nucleotide sequences of miRNAs, 3' UTR sequences of mRNAs, sequence conservation, experimentally validated miRNA target pairs and microarray profile are required. Some useful databases related to miRNA target prediction are summarized in Table **1**.

## 3. EXISTING ALGORITHMS FOR MIRNA TARGET PREDICTION

The supportted organisms and websites of miRNA target prediction algorithms are summarized in Table **2**.

### 3.1 Definition and Problem Formulation

To systematically survey the existing algorithms for miRNA target prediction, we first provided the mathematical definition and formulated the problem of target prediction. For a given miRNA sequence of length *K*, let ***z* = {*z*_1_,...,*z*_*K*_}** denotes its nucleotide composition, where ***z* ε *S***  represents the nucleotide at the *k* th position from its 5' end and *S* = {*A*,*T*,*C*,*G*} . For a testing 3' UTR *m* of an mRNA, a sequence of *N* nucleotides is retrieved from the 3' end of the mRNA and denoted as *s* = {*s*_1_,...,*s_N_*}, where *s_n_* ∈ *S* represents the nucleotide at the ***n*** th position counting from the 3' of the 3'UTR. An illustration of the definition is given in Fig. (**1**). In practice, instead of using the sequence data directly in prediction, important features such as miRNA-mRNA matching pattern and free energy are extracted first to be used for prediction. If let *x* represents a feature vector derived from *z* and *s* with *x_j_* representing the *j* th feature, then the goal of sequence-based target prediction is to decide if mRNA ***m*** is a target based on *x*. From a statistical learning perspective, target prediction is essentially a statistical classification problem. If let *y* ∈ {0,1} represents the status of mRNA *m*, *y* = 1  when mRNA *m* is a target and *y* = 0 otherwise, then the goal is equivalent to identify a function, or a classifier, *f* () that can predict *y*, or, *y* = *f*(*x*). Depending on if training data is available and if *f* is constructed based on statistical learning theory, the approaches can be categorized as either the rule-based or the data driven. The rule-based approaches derive the classifier mainly based on biological prior knowledge and important observations from biological experiments, whereas the data driven approaches rely on training data and formal statistical learning theory. For data driven approaches, define *D* = {(*z*_1_,*s*_1_),...(*z_T_*,*s_T_*)} as a set of *T* training data samples. Naturally, the survey will be carried out according to this two categories. Prior to review the prediction algorithms, we will discuss some important features that have been applied in miRNA target predictions.

### 3.2 Important Features in miRNA Target Prediction

Feature extraction is a crucial element in miRNA target prediction and it will affect sensitivity and specifity of the prediction. Many algorithms share some very critical features that we will discuss in the follwoing sections. Table **3** briefly interprete the features used in different algorithms.

#### 3.2.1. Seed Region Match

In this paper, the “seed” region, which is defined as a sequence from the 1st to 8th nt in the 5' end of the miRNA, has been observed to have high degree of perfect complimentary to the target mRNA sequence. Therefore, nucleotide matching information of the miRNA-mRNA pair in the seed region is considered one of the most important features [28-30]. A depiction of the secondary structure of miRNA binding and seed region is shown in Fig. (**2**). So there exists a few different features extracted from the matching information in the seed region and summarized in the following 7 different types.

- Type 1 [29, 37, 48]: *x* ∈ {0,1} and *x* = 1  if there is perfect  *z*_2_ - *z*_8_ (Watson-Crick) match.
- Type 2 [29, 36]:  *x* ∈ {0,1} and *x* = 1  if there is perfect *z*_2_ - *z*_8_ match with an `A' in mRNA binding with *z*_1_.
- Type 3 [36, 37]:  *x* ∈ {0,1} and *x* = 1  if there is perfect *z*_2_ - *z*_7_ match.
- Type 4 [49]:  *x* ∈ {0,1} and *x* = 1  if there is perfect *z*_1_ - *z*_6_ Watson-Crick or G-U matches and at most one G-U match.
- Type 5 [49]:  *x* ∈ {0,1} and *x* = 1  if there is perfect *z*_2_ - *z*_7_  Watson-Crick or G-U matches and at most one G-U match.
- Type 6 [50]:  *x* ∈{0,1} and *x* = 1  if the number of perfect matches in *z*_1_ - *z*_8_ is more than a cut-off value.
- Type 7 [50]:  *x* ∈{0,1} and *x* = 1  if the number of consecutive perfect matches in *z*_1_ - *z*_8_ is more than a cut-off value.

#### 3.2.2. Conservation

The miRNA is highly conserved across a wide range of species [45], and its targets are also shown to be conserved [45]. When used for target prediction, seed region conservation is often considered due to the importance of seed region. Normally, seed match conservation is defined in the following way [28]: when the same seed match is found in the 3' UTR of one species and also in an orthologous 3' UTR of another species, this seed match is considered to be conserved in this two species.

#### 3.2.3. Free Energy

Free energy refers to the minimum free energy and shows how strong the binding of a miRNA with its target is. Normally free energy is a negative real value and its unit is kcal/mol. The lower the free energy, the firmer the binding structure is and the more likely it suggests the true binding. The free energy of miRNA-mRNA binding is normally assigned by RNAfold program - Vienna RNA Package [51]. Since this program requires a single linear RNA sequence as input, 3' end of the 3' UTR sequence and the 5' end of miRNA sequence are connected by a linker sequence, “LLLLL” [38]. The L is not an RNA nucleotide, thus it does not match with any nucleotide. Given this single linear RNA sequence, Vienna RNA Package will form a structure which has the minimum free energy.

#### 3.2.4. In-Site Features

In addition to the seed region, important features can also be retrieved from other parts of 3' UTR. As showed in Fig. (**2**), the miRNA target binding site is divided into 3 regions: region 5 (seed region), region 3, and total region. Seed region stretches from *z*_1_ to *z*_8_ , region 3 covers  *z*_9_ to *z*_20_ , and total region is defined from  *z*_1_ to *z*_20_ . In this three regions, various features can be calculated including free energy of the corresponding region, the number of matches, mismatches, G:C matches, A:U matches, G:U matches, mismatches, bulges in mRNA, and bulged nucleotides in mRNA.

#### 3.2.5. Accessibility Energy

Accessibility energy represents the open degree of the 3' UTR bounded by a miRNA in the thermodynamic view. The lower the accessibility energy is, the more likely the 3' UTR is to be a target. The unit of accessibility is kcal/mol. Accessibility (**ΔΔ*G***) [34], is defined by the following equation:

(1) $$\Delta \Delta G=\Delta G_{\mathrm{duplex}}-\Delta G_{\mathrm{open}}$$

where Δ*G_duplex_* is the energy gained by the miRNA binding to its targets. Δ*G_open_*  is the energy required to make the target region accessible for miRNA binding and can be calculated as:

(2) $$\Delta G_{\mathrm{open}}=G_{\mathrm{free}}-G_{\mathrm{unpair}}$$

where *G_free_* is the free energy of the ensemble of all secondary structures of the target region. *G_unpair_* is the free energy of all target-region structures in which the target nucleotides are required to be unpaired.

### 3.3. Rule Based Algorithm

Rule based algorithms generally consist of a set of rules to be satisfied by a testing 3' UTR. These algorithms are proceeded by testing the rules according to a particular order. In some cases, the testing order of rules is constrained by the causal relationship of the rules and the possible physical structure of the data. However, since testing a rule is essentially a filtering step, the order of testing the set of the rules will affect the performance of an algorithm. We discussed the following detailed rules of each algorithm according to the order of rules proposed in the papers.

#### 3.3.1. TargetScan and TargetScanS

Both TargetScan [28] and TargetScanS [29] are the algorithmic engine behind the popular TargetScan software, but TargetScan is an early version of TargetScanS. TargetScan is used to predict conserved miRNA targets in mammals. First of all, miRNAs conserved in multiple organisms and a set of candidate orthologous 3' UTR sequences from these organisms are prepared. TargetScan considers both seed match features and the free energy feature. TargetScan searches the 3' UTR for seed match type 1 and disqualify the 3' UTR if no seed match can be identified. If the 3' UTR has seed matches and supposed that *J* seed matches exists, TargetScan increases each one of the *J* 7mer matched region by extending the matching (allowing also G:U pairs) to both sides of the sequence and stops until a mismatch. The basepairing of the remaining part of the miRNA and the 35 nucleotides which are immediately connected to 5' of each seed match in the the 3' UTR is optimized by RNAfold program [51, 52] and a score *Z* of the 3' UTR is computed.

(3) $$Z={\sum^{j}}_{j=1}e^{{-G}_{j}/T}$$

where ***T*** is a preassigned parameter. The ***Z*** scores are calculated for 3'UTRs of each organism. The probing mRNA is predicted to be the target gene if *Z* ≥ *Z_c_* (*Z_c_* is a prechosen threshold) for an orthologous 3'UTR of an organism.

TargetScan was applied to two sets of miRNAs: 79 miRNAs that have homologs in human, mouse, and pufferfish and share identical sequences in human and mouse; 55 miRNAs that have identical sequences in human, mouse and pufferfish. 451 and 115 regulatory target genes are predicted for these two set of miRNAs, respectively. Statistical analysis using shuffled controls indicate that about 30% of predicted mammalian targets are likely to be false positives. 11 of 15 tested targets are experimentally validated. The predicted regulatory targets are enriched for genes involved in transcriptional regulation and a broad range of other functions.

TargetScanS [29] which is a refined or “simplified” version of TargetScan, does not consider free energy and is restricted to predict miRNA targets in mammals, worms and flies. The features of TargetScanS are seed match type 1 or 2 and conservation. A mRNA is predicted as a target only if both features are true. Results show that the false positive rate is reduced to 22% compared to 30% of TargetScan. 5300 human genes (over one third of human genes) are predicted as conserved miRNA targets by TargetScanS.

#### 3.3.2. miRanda

miRanda [31-33] can be used to predict miRNA targets in humans, mice and rats. miRanda consists of two rules to make prediction. This two rules are nucleotide complementariness and binding energy. In the first step, the algorithm aligned miRNA and 3' UTR sequences by using Watson-Crick and G-U match. The scoring matrix is given in Table **4**. Opening gap and extended gap penalty can be assigned by user. A weight parameter is multiplied to the score matrix for different regions of the miRNA to model the different function of 5'end and 3'end of miRNA. Multiple sites can be identified, each with a score reflecting the degree of complementary. The test proceeded to the second step only when the score is greater than a user-defined threshold.

At the second step, Vienna package [53] is used to calculate binding energy for miRNA:sites duplex. 5'end of miRNA and 3'end of potential site are first linked into a single sequence by an 8-bit long linking string formed by character `X' to meet the input format of Vienna package. Secondly, the folding function of Vienna package is called to calculate the free energy of the artificial sequence. Because the characters `X' can not match any characters, the sequence is very likely to form a hairpin structure with an 8-bit loop which consists of `X'. The free energy is also calculated by Vienna package. A site is predicted as a real binding site when its free energy is less than a cut-off value. Additionally, conservation is used to filter out unqualified candidates.

miRanda is applied to predict human miRNA targets. Around 2000 putative human miRNA target are identified, suggesting that less than 10% of the human genes are regulated by miRNAs.

#### 3.3.3. Probability of Interaction by Target Accessibility (PITA)

PITA is used to predict miRNA targets in humans,mice, worms and flies. The key novelty of PITA [34] is the model for the miRNA-target interaction. Such interaction is based on the experimental observation that a strong secondary structure formed by 3' UTR itself will prevent the binding of miRNA.

Based on this observation, a new thermodynamic model for miRNA-target interaction is defined. First of all, the seed match rule is seed match type 3 or *z*_2_ to *z*_8_ match with at most one G:U wobble match. A piece of mRNA sequence is a potential site if it follows the seed match rule. Then the accessibility energy, ΔΔ*G*, of miRNA-site interactions can be calculated as:

(4) $$\Delta \Delta G=\Delta G_{\mathrm{duplex}}-\Delta G_{\mathrm{open}}$$

where Δ*G_duplex_* is the energy of the miRNA binding to the target and Δ*G_open_* is the energy required to make the target region accessible for miRNA binding and can be calculated as:

(5) $$\Delta G_{\mathrm{open}}=G_{\mathrm{free}}-G_{\mathrm{unpair}}$$

where *G_free_* is the free energy of the ensemble of all secondary structures of the target region. *G_unpair_* is the free energy of all target-region structures in which the target nucleotides are required to be unpaired. Furthermore, the score of a 3' UTR containing multi-sites can be calculated as

(6) $$T=\log\left(\sum_{i=1}^{n}e^{s_{i}}\right)$$

where *n* represents the number of candidate target sites in the 3' UTR and *S_i_* represents the ΔΔ*G* for *i* th site.

#### 3.3.4. DIANA-microT

“DIANA-microT” is proposed in [54] as an approach to predict human miRNA targets. DIANA-microT retrieves orthologous human and mouse 3' UTRs from mRNA Reference Sequences (RefSeq) database and 94 miRNAs conserved in human and mouse. A window of 38 nucleotides is slid one nucleotide at a time across a orthologous 3' UTR to form a set of overlapping 38-nt long segments in the 3'UTR. DIANA-microT applies a modified dynamic programming algorithm to determine the minimum free energy for each segment with a miRNA. Then, the following features are examined:

1. *x*_1_ ∈ {0,1} and *x*_1_ = 1 if there exists 3 consecutive WC matches.
2. *x*_2_ ∈ {0,1} and *x*_2_ = 1 if the free energy is lower than a user defined threshold.
3. *x*_3_ ∈ {0,1} and *x*_3_ = 1 if from *z*_1_ to *z*_10_, there are more than 7 WC matches or G-U matches; however, the number of G-U matches cannot be less than 2 and each of the G-U match must be surrounded by 2 WC matches; moreover, only one bulge is allowed, which must also be surrounded by the WC matches longer than the length of the bulge.
4. *x*_4_ ∈ {0,1} and *x*_4_ = 1 if from *z*_8_ to *z*_15_, there exists at least one loop or bulge and it should be either 2 to 5 nucleotides long if on miRNA side or 6 to 9 nucleotides long if on mRNA side.
5. *x*_5_ ∈ {0,1} and *x*_5_ = 1 if from *z*_15_ to *z*_22_, there are more than 5 WC or G-U matches and exists at most a single-nucleotide or dinucleotide bulge, provided that it is surrounded by two or three base-paring, respectively.

A 3' UTR is predicted as the target of a miRNA or *y* = 1 only if 3' UTR has one segment for which all the features *x*_1_,..., *x*_5_ are equal to 1. DIANA-microT successfully identified all of the documented *C. elegans* miRNA-target pairs and seven predicted mammalian miRNA targets are validated experimentally.

#### 3.3.5. RNAhybrid

RNAhybrid, proposed in [48], is a program that predicts multiple potential binding sites of miRNAs in large 3' UTRs. RNAhybrid utilizes seed match, free energy, and *p*-value of the free energy estimation as features. The default seed match feature is the seed match type 1 but user defined seed matches are allowed as well. Given a miRNA and a 3' UTR, RNA-hybrid will find all possible binding structures starting with the seed match in the 3' UTR and pick the structure which gives the minimum free energy (MFE). MFE is used as the second feature and its p-value is used as the third feature. Finally, a 3' UTR is predicted as the target of a miRNA (*y* = 1) if both MFE and the p-value are less than user defined cutoffs. RNAhybrid was applied to predict Drosophila miRNA targets in 3' UTRs and coding sequence. Most of the perviously predicted miRNA targets can be found by RNAhybrid.

#### 3.3.6. MicroInspector

MicroInspector is presented in [49] as a scanning software for detecting miRNA binding sites. MicroInspector program generates a list of possible target sites, sorted by free energy values. The prediction is based on four features. The first feature (*x*_1_) is the seed match type 4 or 5. After finding the seed matches, MicroInspector extracts a 32-nt sequence in 3' UTR starting from the seed matches. Subsequently, the binding structure and free energy are predicted by hybridization folding algorithm [48]. The second feature (*x*_2_) is free energy: *x*_2_ = 1 if the free energy is less than a cut-off value, otherwise *x*_2_ =0. The third feature (*x*_3_) is *x*_3_ = 1 if  *z*_16_ - *z*_21_ of the binding structure has less than 2 mismatches, otherwise *x*_3_ = 0. The fourth feature (*x*_4_) is self-complementarity: *x*_4_ = 1 if miRNA 3' UTR has no self-complementarity, otherwise *x*_4_ = 0. Then 3' UTR is predicted as the target of miRNA (*y* = 1) if all the features are true. This program successfully found all the known miRNA-target interactions.

#### 3.3.7. MovingTargets

MovingTargets [50] is a program that predicted miRNA target in Drosophila. To perform the prediction, 3' UTR sequences that are more than 12 nt long and at least 80% conserved between D. melanogaster and D. pseudoobscura are obtained. If the 3' UTR is longer than 50 nt, a 50 nt long window will slide across the 3' UTR. The window starts from 5' and shifts 5 nt at a time towards the 3' end of 3' UTR. The binding structure is predicted by M. Zuker's DINAMelt Server software [55] for miRNA and each window. Prediction is made based on 4 features. The first feature (*x*_1_) is free energy: *x*_1_ = 1, if the free energy of this binding structure is less than a cut-off value, otherwise, *x*_1_ = 0. The second feature (*x*_2_) is seed match: *x*_2_ = 1, if there exists a seed match type 6, otherwise, *x*_2_ = 0. The third feature (*x*_3_) is also a seed match: *x*_3_ = 1, if there exists a seed match type 7, otherwise, *x*_2_ = 0. The fourth feature (*x*_4_) is the number of G:U matches: *x*_4_ = 1, if the number of G:U matches from *z*_1_ to *z*_8_ is less than a cut-off value, otherwise, *x*_4_ = 0. A potential binding site is predicted if all the features are true. A 3' UTR is predicted to be a target if it has more than user defined number of potential binding sites. Three of predicted candidates were tested and all of them are experimentally verified.

#### 3.3.8. Nucleus

“Nucleus” [56] is a computational model for miRNA target site recognition in Drosophila. The process of prediction of Nucleus starts with finding the best weight for GC, AU, and GU matches (*w_GC_* = 5, *w_AU_* = 2, *w_GC_* =0) based on 25 experimentally validated training set. A score for the seed region from *z*_1_ to *z*_8_ is then assigned, which is the weighed sum of consecutive base pairs being either GC, AU, or GU. The prediction is then made based on two features. The first feature (*x*_1_) is the score of the seed: *x*_1_ = 1, if the score of the seed is larger than a cut-off value, otherwise, *x*_1_ = 0. After finding the binding sites, a window of 40 bases (started from the seed region) is extracted from the 3' UTR and binding structure is predicted using the MFOLD RNA folding program [57]. The second feature (*x*_2_) is *x*_2_ = 1, if the free energy of the binding structure is less than a cut-off energy, otherwise, *x*_2_ = 0. A 3' UTR is predicted as the target of a miRNA (*y* = 1) only if both features *x*_1_ and *x*_2_ are true. Nucleus was applied to a set of 74 Drosophila melanogaster miRNAs and prediction was conducted among conserved 3' UTR sequences in fly mRNAs. It is found that many key developmental body patterning genes such as hairy and fushi-tarazu are likely to be transactionally regulated by miRNAs.

### 3.4. Data Driven Algorithms

#### 3.4.1. PicTar

PicTar [37] is method that predicts miRNA targets in vertebrates, flies, and nematodes. Input of PicTar is a set of coexpressed miRNAs and sets of orthologous 3' UTRs. To compile the training dataset, PicTar first records the positions that satisfy “seed match type 1 or 3” in all 3' UTRs. Secondly, it checks whether perfect seed matches are conserved or not, which means the same miRNA binds to the overlapping aligned positions in the 3' UTRs of the orthologous mRNAs of all species under consideration. If the perfect matches are conserved, PicTar further checks if optimal miRNA - target binding free energy predicted by RNAhybrid [48] is below a cutoff value. Perfect matches that pass these steps are called anchors. A 3' UTR containing a sufficient number of anchors is considered as a candidate. Each candidate 3' UTR is searched separately for sites with perfect matches (seed match type 1 or 2) and imperfect matches. Insertions or mutations in the mRNA sequence of a perfect matches (G:U pairs are not allowed) are allowed as long as its free energy of binding blew a cutoff value, which is predicted by RNAhybrid [48]. Subsequently, sites with imperfect matches have to pass a free energy filter that filters out sites with free energy larger than two-thirds of that of miRNA-mRNA duplex with the perfectly match. As a result, most of the sites with imperfect match will be removed. Sites with perfect matches might also be subject to a free energy filter but with a larger cut-off. The remaining candidate 3' UTRs are used as training data set.

A Hidden Markov Model (HMM) is then built to model the fact that several different miRNAs can act together to repress the same gene. Particularly, it is assumed that the 3' UTR of a gene is generated by the HMM, whose states are target sites of coexpressed miRNAs plus the background nucleotide sequence. Given *M* states reflecting the total number of different miRNAs that had combinatorial regulatory effect, a target 3' UTR sequence can be generated in the following way: at each step one of the states is chosen with transition probabilities *ρ_i_* for *i* = 0 to *M*, where *ρ_o_* is the transition probability of background. Depending on the nature of the state, a certain sequence will be emitted. When a miRNA target site state is chosen, the 7-mer or 8-mer sequence representing the binding site of the miRNA will be emitted. Note that the emitted binding site could be either perfect matching with the miRNA seed region (with the probability p, say *p* =0.8) or imperfect matching (with probability 1- *p*). Otherwise, in the background state, one nucleotide will be emitted. Background is modeled with the Markov model of order 0. This model is then trained using Baum-Welch algorithm [58] based on the training data set.

To perform the prediction, PicTar computes the log ratio of the probability of the probing sequence being generated by this HMM model versus the probability that it is generated by the background process alone. This score also reflects the likelihood that the probing 3' UTR is targeted by a set of coexpressed miRNAs. The final score of the sequence is the average of the PicTar scores for all orthologous 3' UTRs that are used to define anchor sites. A 3' UTR is predicted as the target if this final score is larger than a cut-off value. PicTar was applied to search targets in C. elegans that are conserved in 3 nematodes. The result shows that more than 10% of C. elegans genes are predicted as miRNA targets and miRNAs regulate biological processes through targeting genes that are functionally related to each other.

#### 3.4.2. miTarget

miTarget is a machine learning based algorithm [38, 59]. Due to the fact that the mechanism of a miRNA binding to their targets is still poorly understood, the advantage of miTarget is that algorithm can obtain useful information from training data instead of using artificial rules as filters. To build the training data set, 152 positive targets and 83 negative targets are collected from the literature [38]. 163 negative targets are inferred from miRNA let-7 on mRNA lin-41 [60] and lin-28 [61]. A miRNA sequence and a potential target sequence are linked together with a linker sequence, “LLLLLL”, to form a binding structure by RNAfold program - Vienna package [53]. As showed in Fig. (**2**), the miRNA target binding site is divided into 3 regions: region 5 (seed region), region 3 and total region. Seed region stretches from *z*_1_ to *z*_8_, region 3 covers *z*_9_ to *z*_20_ and total region is defined from *z*_1_ to *z*_20_. Position-based features are matching status of 20 positions of the total region. Structural features are the numbers of matches, mismatches, G:C matches, A:U matches, G:U matches and other mismatches of these three regions. In addition, thermodynamic features are the free energy of these 3 regions which are also calculated by Vienna package [53]. Consequently, a miRNA-site duplex is represented as a feature vector with 41 features. A SVM [62, 63] with RBF kernel is trained based on the training data and the feature vector. miTarget do not consider conservation information to avoid the loss of sensitivity, on the other hand, the false positive rate is increased.

miTarget predicted significant functions of human miRNA miR-1, miR-124a and miR-373 using Gene Ontology (GO) analysis and unveiled the importance of pairing positions *z*_4_, *z*_5_ and *z*_6_ of a miRNA in a feature selection experiment.

#### 3.4.3. mirTarget

MirTarget is another SVM based algorithm published in [35, 36]. In this algorithm, microarray data [2] which includes two cell lines are used to generate the training data. A gene is defined as a positive target gene if its expression level is reduced, when compared with mock transfection, by at least 40% with a p-value < 0.001. On the contrary, a gene is a negative target if its gene expression level is from 95% to 120% with a p-value > 0.3 in both cell lines.

A feature vector with 113 features is defined for a miRNA and target pair. 20 nucleotides around the seed in 3'UTR are defined as local context. 3' UTR sequences from human genes orthologs in mouse, rat, dog, and chicken are analyzed to identify miRNA seed matches, and the level of seed conservation is recorded as seed conservation feature. Other features are derived as: 6 seed match type features including seed match 2 and type 3, 20 base position features including single nucleotide (A,T,C,G) and dinucleotide (AT,AA,TG...), 80 position features in the local context (each position has 4 options, 4*20 = 80 ), 17 additional position features (such as Position 11 A or U), 7 other features including accessibility and location of the binding site. Considering that some 3' UTRs have multiple sites, the authors also developed a scoring system to assign a score to the 3' UTR using the formula

(7) $$\mathrm{Score}=100^{*}\left(1-\sum_{i=1}^{n}p_{i}\right)$$

where *n* represents the total number of candidate target sites in a 3' UTR and *p_i_* represents the statistical significance p-values for each of these candidate sites as estimated by SVM [64].

MirTarget observed that about half of the predicted miRNA target sites in human are not conserved in other organisms. The algorithm has been validated with independent experimental data for its improved performance on predicting a large number of miRNA downregulated gene targets.

#### 3.4.4. RNA22

RNA22 is presented in [65] as a method for identifying miRNA binding sites and their corresponding heteroduplexes. To construct the training data, 644 mature miRNA sequences are analyzed to remove near-duplicate entries end, which end up with 354 miRNA sequences. The Teiresias algorithm [66] is then applied to discover patterns in this set of the miRNA sequences. The criterions used in the Teiresias algorithm for pattern searching are that a pattern must be longer than 4, at least 30% of the positions of a pattern can be specified, and each pattern has to appear at least twice in 354 miRNAs. An example of such pattern can be [AT][CG].TTTTT[CG]G..[AT], which represents all instances that have their first position occupied by either an A or T, their second position by a C or G, their third position by any nucleotide, their fourth position by a T, etc.

The frequency of any kinds of trinucleotides is then calculated based on the training data. A trinucleotide-sequence is a sequence including any three nucleotides and 0 to 20 long dots (undecided nucleotides), AC..G, CA....................T etc. For the calculation, a second-order Markov chain is assumed and the times of appearance of each pattern in the genomic data are counted. Let us use an example pattern (A..[AT].C..T...G) to explain this approach. Due to the Markov chain, the probability of any pattern appeared in this genomic data can be obtained as

(8) $$P\left(A..\left[\mathrm{AT}\right].C..T..G\right)=P\left(C..T...G|A..\left[\mathrm{AT}\right].C..T\right)P\left(A..\left[\mathrm{AT}\right].C..T\right)=P\left(C..T...G|C..T\right)P\left(\left[\mathrm{AT}\right].C..T|A..\left[\mathrm{AT}\right].C\right)P\left(A..\left[\mathrm{AT}\right].C\right)=P\left(C..T..G|C..T\right)P\left(\left[\mathrm{AT}\right].C..T|\left[\mathrm{AT}\right].C\right)P\left(A..\left[\mathrm{AT}\right].C\right)=P\left(C..T...G|C..T\right)P\left(\left[\mathrm{AT}\right].C..T|\left[\mathrm{AT}\right].C\right)P\left(A..\left[\mathrm{AT}\right].C|A..\left[\mathrm{AT}\right]\right)P\left(A..\left[\mathrm{AT}\right]\right)$$

which can be estimated as

(9) $$P\left(A..\left[\mathrm{AT}\right].C..T...G\right)\approx \#\left(C..T..G\right)/\left(\#\left(C..\mathrm{TA}\right)+\#\left(C..\mathrm{TC}\right)+\#\left(C..\mathrm{TG}\right)+\#\left(C..\mathrm{TT}\right)\right)=\#\left(\left[\mathrm{AT}\right].C..T\right)/\left(\#\left(\left[\mathrm{AT}\right].C..A\right.\right)+\#\left(\left[\mathrm{AT}\right].C..C\right)+\#\left(\left.\left[\mathrm{AT}\right].C..G\right)+\#\left(\left[\mathrm{AT}\right].C..T\right)\right)=\#\left(A..\left[\mathrm{AT}\right].C\right)/\left(\#\left(A..\left[\mathrm{AT}\right].A\right.\right)+\#\left(A..\left[\mathrm{AT}\right].C\right)+\#\left(A..\left[\mathrm{AT}\right].G\right)+\#\left(\left.A..\left[\mathrm{AT}\right].T\right)\right)$$

where # represents the number of appearance in the training data. Patterns of higher probabilities are more significant patterns in this training data and therefore patterns with log-probability lower than -38 are discarded. In the end of this stage, 233,554 miRNA patterns remain. It is then assumed that if a small piece (36 nucleotides long) 3' UTR contains a lot of significant patterns, this small part is very likely to be a binding site of miRNAs. RNA22 calls this 36 nucleotides long region as “target island” when it contains at least 30 patterns. The reason for choosing 36 as the length of a target island is because that the binding site is usually less than 36 nucleotides. It should be noted that these target islands are decided only by patterns without reference to any specific miRNA.

To predict the target of a miRNA, binding structures of this miRNA with target islands of candidate 3' UTR are formed and the folding energy is calculated by Vienna package [53]. Three features are considered

1. *x*_1_ ∈ {0,1} and *x*_1_ = 1 if W-C pairs between miRNA and target island is more than a cut-off value.
2. *x*_2_ ∈ {0,1} and *x*_2_ = 1  if the number of mismatches in *z*_1_ - *z*_8_ is lower than a cut-off value.
3. *x*_3_ ∈ {0,1} and *x*_3_ = 1 and  if the folding energy is lower than a cut-off value.

A binding site is predicted to be a target if all features are equal to 1.

226 targets predicted by RNA22 were tested in a luciferase reporter gene assay and 168 of them are observed to be observed miRNA-dependent repression.

#### 3.4.5. SVMicro

SVMicro [67] is the third SVM based target prediction algorithm. Most published miRNA target prediction algorithm focused on modeling the interaction between miRNA and targeted site but seldom worked on building model for interaction of miRNA and target 3' UTR. SVMicro is a two-stage SVM based method that models the mechanism of how miRNA binds to a site as well as how miRNA target a 3' UTR.

To prepare the training data, experimentally validated miRNA-site and miRNA-UTR pairs are obtained from TarBase 4.0 [1] as positive training data. Negative miRNA-UTR pairs are extracted also from Linsley's experiment [2] but using up-regulated genes whose expression levels are greater than 1.2 fold and the p-value is greater 0.2. Additionally, a set of seed matching rules, which base on the observation of real binding structure in TarBase, are designed to select potential binding sites from 3' UTR sequence with minimal loss of real target site.

A vector of 111 features is designed for site-SVM to predict whether a site is a potential binding site of miRNA. To this end, first of all, seed match type, which includes 6mer, 7mer-A1, 7mer-m1, 7mer-m8 and 8mer, is recorded as 5 seed type features. Secondly, nucleotide matching status and 2-mer matching status of from *z*_1_ to *z*_20_ are recorded as 39 position specified features. Thirdly, the entire binding structure is divided into seed region, 3'region and total region. Free energy and the number of matches, mismatches, G:U wobbles, gaps, bulges in mRNA and bulged nts in mRNA of each region are collected to form another 21 regional features. Fourthly, the accessibility energy of site is calculated. Fifthly, the content of nucleotides and 2mers of the context of both side of seed are calculated as 40 context features. Finally, the number of homologous 3' UTRs, seed conservation score, site conservation score, context conservation score are analyzed as 4 conservation score. After training, a score is assigned to each site by site-SVM. The larger the score, the more likely the site is a real site.

After site prediction, 3' UTR SVM, with a 27-feature-vector, is designed to decide whether the entire 3' UTR is a target of a miRNA. The length of 3' UTR and top site scores are collected as two features. Density and partial (within 100nts) maximum number of potential sites as well as positive sites are recorded as 4 sites density features. The number of potential sites, positive sites and top score of all sites, *z*_2_ - *z*_7_ match sites, *z*_1_ - *z*_7_ match sites, *z*_2_ - *z*_8_ match sites, *z*_1_ - *z*_8_ match sites, and other type of site are formed as the remaining 21 features.

#### 3.4.6. TargetBoost

TargetBoost [68] is proposed to predict if a up to 24- *nt* long site from a 3' UTR region is a target site of a given miRNA in C. elegans and D. melanogaster. The central idea underlying TargetBoost is to find differential DNA nucleotide sequence patterns from training data, which can best discriminate true and false target sites. However, it is different from the other surveyed algorithms in the sense that it incorporate neither prior knowledge about miRNA binding nor energy information into the procedure of searching for the pattern. The classification algorithmic engine behind TargetBoost is the boosting genetic programming algorithm, or GPboost. GPboost identifies the differential patterns using genetic programming (GP) [69, 70] in a boosting paradigm and the assembles the prediction from each pattern into the final prediction. The feature set ${\left\{x\right\}}_{J=1}^{j}$ here is a set of 24- *nt* long sequence patterns, which also include gaps. The GPboost classifier assumes the standard form of the boosting classifier as

(10) $$f\left(S_{1:N}\right)=\mathrm{sign}\left(\sum_{j=1}^{J}\alpha_{j}h\left(S_{1:N},x_{j}\right.\right)$$

where *h*(*S*_1:*N*_,*x_j_*) is a classifier that predicts 1 if *S_*1*:N_* conforms to the pattern *x_j_* and -1, otherwise. *α_j_* is the weight on the prediction of *h* based on the *j* th pattern feature. The algorithm for learning the classifier (10) from the training data proceeds as follows

The Targetboost Algorithm Set $w_{t}=1/T\forall n$ and *f*_o_(*S*_1:*N*_) = 0

Iterate for *j* =1 to *J*

identify the *j* th feature pattern *x_j_* by

(11) $$x_{j}=\arg\;\min_{x}\;\sum_{t=1}^{T}w_{t}|h\left(s_{t,1:N},x\right)-l_{t,c}|,$$

Compute *α_j_* that minimizes a loss function *L*

(12) $$\alpha_{j}=\arg\;\min_{\alpha }\sum_{t=1}^{T}L\left(l_{c},f_{j-i}\left(s_{t,1:N}\right.\right)+\alpha h\left(\left.s_{t,1:N},x_{j}\right)\right)$$

Set *f_j_*(*s*_1:*N*_) = *f*_*j*-1_(*s*_1:*N*_)+ *α_j_h*(*s*_1:*N*_,*x_j_*); Update $w_{t}\forall t$ by $w_{t}=\partial /\left[\partial f_{j}\right]L\left(l_{c},f_{j}\left(s_{1:N}\right),\left.x_{j}\right)\right)$.

To solve the minimization of (11), genetic programming is applied based on a set of sequence matching criterions and the concept of evolutionary algorithms. The loss function is chosen to be the exponential loss but with regularization introduced to account for noise or outliers and the overall scheme can be considered as the regularized AdaBoost.

The Targetboost is trained and tested on a data set consisting of 36 experimentally validated true target sites and a large number of random sequence as negative sites. The performance was shown to be slightly better when compared with two other rule-based algorithms, RNAhybrid and Nucleus. Examining the obtained patterns reveals the tendency to have near-perfect complementary at the 3' end of target sites, a fact consistent with the current consensus about miRNA target. Targetboost was also applied to search the target sites of 78 *D. melanogaster* miRNAs and the similarity and difference in the prediction results with RNAhybrid were studied. The key feature of Targetboost is that it is not constrained by, for instance, seed region complementary, which, however, can be considered to be both advantage and disadvantage since it has potential to produce more true positives but at the price of increasing false positives.

### 3.5. Algorithm Using Expression Level Data

GenMiR++ [71] is a Bayesian algorithm that predicts targets based on expression profile of mRNA and miRNAs. In addition to the expression profile, a list of candidate targets predicted by a sequence-based algorithm such as TargetScan [29] needs to be provided. GenMiR++ is designed to further predict which candidate targets are *bona fide* functional targets. For this purpose, a Bayesian generative model is built to reflect assumed regulatory effect of miRNAs on targets. To this end, it is first assumed that mRNAs share a common background expression level within a specific tissue. Secondly, the expression level of a target mRNA is assumed to be down-regulated and the degree of down-regulation is due to the linear combinatory effect of the regulatory miRNAs. Now given *G* candidate mRNAs and *K* miRNAs, let *e_gt_*, *v_kt_* and *µ_t_* represent the respective expression levels of mRNA *g* , miRNA *k*, and background in tissue *t* and *v_t_* = [*ν*_1*t*_,...,*ν*_*kt*_]^T^. Then these assumptions are formulated by the following Gaussian likelihood function

(13) $$p\left(e_{\mathrm{gt}}|\mu_{t},\beta_{g},\lambda ,\gamma_{t},\nu_{t},\sigma_{t}^{2}\right)=N\;\left(\mu_{t}-\gamma_{t},\lambda B_{g}v_{t},\sigma_{t}^{2}\right)$$

where *β _g_*ε {0,1}^*K*×1^ is a *K*×1 vector of indicators, whose *k* th element *β_gt_* is 1 if gene *g* is a target of miRNA *k* and 0, otherwise, $\lambda \in R_{+}^{K\times 1}$ is a vector of some positive regulatory weights of the *K* miRNAs, *B_g_* = *diag*(*β_g_*), *γ_t_* is a positive tissue scaling parameter accounting for the difference in tissue specific hybridization conditions and expression normalization, and $\sigma_{t}^{2}$ is the variance of the Gaussian model. Given the expression levels of mRNAs *g* and *K* miRNAs in all *T* tissues, the goal of prediction is to infer the values (0 or 1) of the indicators $\beta_{g}\forall g$ . Note that $\mu_{t},\lambda ,\gamma_{t},\sigma_{t}\forall t$ are unknown model parameters to be estimated. Under a Bayesian framework, the prior distributions needs to be specified for all the unknowns. To this end, the conjugate exponential family Gaussian and Gamma priors are adopted, which introduced additional hyper-parameters *θ* to be estimated. For *β_g_*, the prior distribution reflects the prediction results of the sequence-based algorithm. Let *c_gk_* ∈ 0,1 be an indicator such that *c_gk_* = 1 denotes gene *g* is predicted by the sequence-based algorithm as a target of miRNA *k* and *c_gk_* = 0, otherwise. Then, *p*(*β_gk_* = 0| *c_gk_* = 0)= 1 since the genes not predicted by the sequence-based algorithm are not even in the candidate target list. Further, it is defined that

(14) $$p\left(\beta_{\mathrm{gk}}=1|c_{\mathrm{gk}}=1\right)=\pi$$

where *π* is an unknown probability to be estimated. Once the likelihood function and the prior are formulated, the goal is to obtain an estimate of $\beta_{g}\forall g$ from the posterior distribution $p\left(\beta_{g}|e_{\mathrm{gt}},v_{t},c_{g,k},\forall t\&k\right)$. Given the high complexity of the model, the posterior distribution cannot be obtained analytically. A variational Bayesian Expectation Maximization (VB-EM) algorithm is proposed to numerically approximate the distribution.

GenMiR++ was applied to the expression data of 151 human miRNAs and 16,063 mRNAs across a mixture of 88 normal and cancerous tissue samples. A candidate list of 114 miRNAs and 890 mRNAs were obtained using TargetScanS. GenMiR++ identified a total of 6,387 miRNA-target pairs and a subset of 1,597 target pairs for 104 human miRNAs with high confidence. Experimental validation was performed on the predicted high confidence targets of miRNA *let* – 7*b* to exam its misregulation in retinoblastoma. Quantitative real-time PCR was performed to measure the mRNA abundance of the predicted let-7b targets. A list 34 targets predicted by TargetScan was considered, among which 12 were predicted by GenMiR++ to be high confidence targets. The PCR experiments showed that 5 out of 12 (42%) high confidence targets were down-regulated whereas only 2 out of rest of 22 (99%) TargetScanS predictions were down-regulated. This represented an increase of prediction specificity but with only a little reduction of sensitivity.

## 4. PERFORMANCE COMPARISON OF DIFFERENT ALGORITHMS

We investigated the importance of features and tested the performance of a few surveyed algorithm using experimentally validated targets.

In order to obtain the positive testing data, only experimentally validated targets are considered. Targets sequences are downloaded from TarBase [1], a database that records experimentally validated targets of several species. Alignment of target sequences with the respective genomes is performed to examine the validity of these records; a target is excluded if the perfect alignment cannot be achieved. In the genome, 118 positive miRNA-UTR pairs are retrieved.

To obtain the negative miRNA-UTR pairs, microarray experimental data of Linsley's study [2] is analyzed. In that study, multiple miRNAs are transfected in cell lines and the global effect of miRNA overexpression is examined by microarray. Two cell lines (HCT116 Dicerex5 and DLD-1 Dicerex5) are included in the Linsley's study and global gene expression profiles are collected to evaluate expression changes due to miRNAs transfection. Probe IDs are mapped to RefSeq IDs with NCBI gene index files, and multiple probe signals for the same gene are averaged to represent the expression level of the gene. For a specific transfected miRNA, the mRNA is considered as a negative target if its expression is larger than 1.2 fold of that in the mock transfection experiment and at the same time p-values must be less than 0.03 in both cell lines. Nine miRNAs from the Linsley dataset, hsa-let-7c, hsa-miR-15a, hsa-miR-16, hsa-miR-17-5p, hsa-miR-192, hsa-miR-20, hsa-miR-215, hsa-miR-103 and hsa-miR-106b are selected for modeling, training and testing. Finally, 278 miRNA-UTR pairs are included as negative data. Sequences of all 3' UTRs are obtained from NCBI. All sequences of miRNAs are retrieved from miRBase 10.1.

First, we evaluated the marginal distribution of features in the form of histogram in both positive and negative data sets. Even though the marginal distributions cannot reveal combinatory discriminative importance of features, they provide information about the discriminative power of each individual feature. Fig. (**3**) shows the histograms of 12 different features. In each sub-figure, the *x* axes represents the feature values and the *y* axes denotes the relative frequency (probability). Histograms from the negative and positive data are represented by the black and white bars respectively. The names of the 12 features are labeled beneath each sub-figure. It is clear that all three seed match type features as well as the number of matches in seed region all have good discriminative power. The free energy and accessibility energy features show relatively good discriminative potential. However, the features including the number of mismatches and GU matches in the binding site do not appear to be important features for target prediction.

Next, we evaluated the Receiver Operating Characteristic (ROC) performance of several different algorithms of both rule-based and data-driven categories including targetScan, miRanda, Pita, SVMicro and RNAhybrid. The reasons for choosing these algorithms for testing are: first, they are representative in each categories, and secondly, softwares of some other algorithms are not publicly available. ROC performance is normally evaluated as a plot of *sensitivity* vs. 1- *specify*, where

(15) $$\mathrm{sensitivity}=\mathrm{TP}/\left(\mathrm{TP}+\mathrm{FN}\right)$$

and

(16) $$\mathrm{specifity}=\mathrm{TN}/\left(\mathrm{TN}+\mathrm{FP}\right)$$

where TP stands for true positive, TN stands for true negative, FN stands for False negative, and FP stands for False Positive. *Sensitivity* is also called true positive rate, 1– *specifity* represents the false positive rate.

The existing algorithms targetScan, miRanda, Pita, SVMicro and RNAhybrid are tested on the testing data set and the ROC curves are shown in Fig. (**4**). The conservation in targetScan and miRanda are not considered in this test. In targetScan, if any potential site passes the rule of perfect 8-mer, 7mer-m8, or 7mer-1A match for a miRNA, the whole 3' UTR will be predicted as the target. When the decision threshold for one algorithm cannot be changed such as TargetScan, the result of ROC curve will be a point. For all other algorithms, when altering the threshold, different sensitivity and specificity can be obtained and a complete curve instead of a point can be drawn. Area Under the Curve(AUC) of each algorithm is calculated to measure the performance of the algorithm. The higher the AUC, the better the algorithm. As can be seen, SVMicro has the overall best performance in term of AUC, which should be expected since it considers a variety of features in prediction. TargetScan has relatively good sensitively but produces high false positives. For a small false positive rate, Pita can achieve relatively high sensitivity than RNAhybrid. This could be due to the inclusion of accessibility feature in Pita. However, the performance of miRanda becomes comparable with RNAhybrid at high false positive rate.

## 5. CONCLUSION

In this paper, we surveyed a large number of existing computational algorithms for miRNA target predictions. The survey is carried out according to the two categories of the target prediction algorithms - the rule-based and the data driven approaches. In Tables 2 and 3, we summarized the information of each algorithm including their supported organism, websites, approaches, etc. To evaluate the performance of a few representative algorithms, a testing data set including experimentally validated positive miRNA targets was constructed. Histograms of different features and ROC performance of each algorithm were evaluated. The histograms confirm the current consensuses on the importance of seed region and energy in target prediction. The ROC curve also reveals that utilizing more information makes the algorithm have better performance.

Despite the recent advances and the initial impact of these algorithms on the miRNA target research, key problems still exist that prevent the computational approach from playing more active role in target prediction. Mainly, these algorithms tend to produce an excessively large number of false positives, thus still unable to generate meaningful, workable hypotheses for subsequent experimental testing. Poor understanding of miRNA targeting mechanism is partially to be blamed and the rules derived from experimental observation are not adequately specific.

To this end, data driven algorithms hold the potential to uncover important features that might not be obviously observed. However, these approaches are limited at this stage mainly by the lacking of both experimentally validated positive and negative targets data. New emerging databases such as MiRecord will be essential for releasing the full potential of data driven algorithms. With the increasing experimentally validated positive and negative data, we expect high impact of these data on the overall research of computational miRNA targets prediction. Another problem with current algorithms is that the majority only utilizes the sequence information. Although increasing attention has been given to include microarray data with miRNA overexpression for target prediction, researches in this front are still new. In addition, data generated from the IP pull down of RISC [72-75] and large scale proteomic study of miRNA addition and deletion [76, 77] also provides high quality knowledge about the direct miRNA-target interaction. So far, only the IP pull-down data of [75] for C. elegans has been investigated in [78] and the others especially for human has not been considered. No attempt of incorporating data from proteomic study has been reported. As a result, to further improve the performance of miRNA targets prediction, especially for genome-wide prediction, the systems biological approach that integrate multiple levels of relevant data as well as the pathway and networks information is the path to follow and will be the focus of this research for the years to come.

## Figures and Tables

**Fig. (1):**
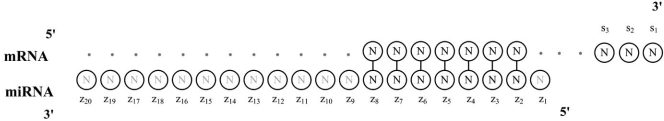
An illustration of the definition of a miRNA and its target mRNA.

**Fig. (2):**
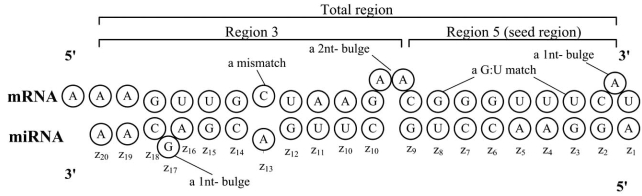
An illustration of the secondary structure of miRNA-mRNA paring.

**Fig. (3):**
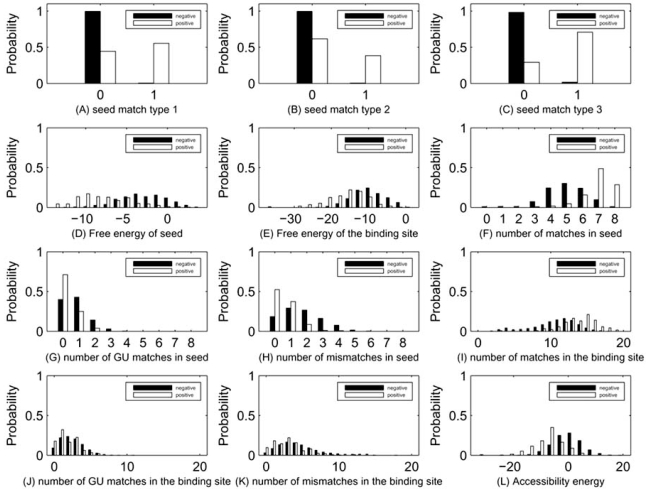
Histograms of different features.

**Fig. (4):**
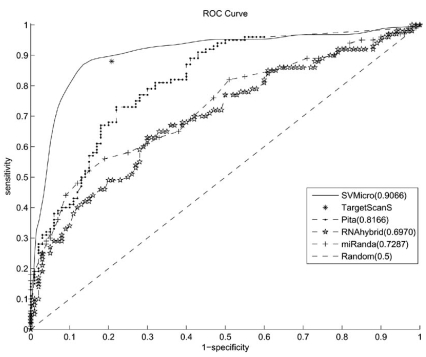
The ROC curve of different algorithms.

**Table 1:** Online Resource for miRNA Target Prediction

Category	Website
Genome of different species	NCBI FTP(ftp://ftp.ncbi.nih.gov/genomes/) UCSC FTP(ftp://hgdownload.cse.ucsc.edu/goldenPath/
Homologous gene information	UCSC (http://genome.ucsc.edu/) NCBI(http://www.ncbi.nlm.nih.gov/sites/entrez?db=homologene)
Sequence and information of miRNAs	miRBase(http://microrna.sanger.ac.uk/sequences/index.shtml)
Experimentally validated miRNA targets	TarBase(http://diana.cslab.ece.ntua.gr/tarbase/) miRecords(http://miRecords.umn.edu/miRecords)
Computational predicted targets	miRecords(http://miRecords.umn.edu/miRecords)

**Table 2:** Support Organisms and Websites of miRNA Target Prediction Algorithms

Name of the Program	Supported Organisms	Website
TargetScanS	Mammals, worms, flies	http://www.targetscan.org/
miRanda	Humans, mice, rats	http://www.microrna.org/microrna/ releaseNotes.do
PITA	Humans, mice, flies, worms	http://genie.weizmann.ac.il/pubs/m ir07/mir07_browse.html
DIANA-microT	Humans	http://diana.cslab.ece.ntua.gr/
RNAhybrid	Any	http://bibiserv.techfak.unibielefeld.de/rnahybrid/
microInspector	Any	http://www.imbb.forth.gr/microinspector/
MovingTargets	Flies	Available on DVD by request
Nucleus	Flies	N/A
PicTar	Nematodes, vertebrates, flies	http://pictar.mdc-berlin.de/
miTarget	Any	http://cbit.snu.ac.kr/~miTarget/
mirTarget	Any	N/A
rna22	Any	http://cbcsrv.watson.ibm.com/rna22.html
SVMicro	Any	N/A
Targetboost	Worms, flies	https://demo1.interagon.com/targetboost/
GenMiR++	Any but require both miRNA & mRNA expression profile	http://www.psi.toronto.edu/genmir/code/

**Table 3:** Features of miRNA Target Prediction Algorithms

Name of the Program	Features of Different Algorithms			Approach
	Seed Match	Free Energy	Conservation	Rule Based
TargetScan	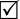	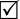	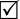	Rule based
TargetScanS	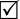		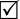	Rule based
miRanda		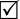	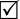	Rule based
Pita	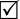	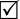		Rule based
DIANA-microT	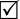	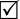		Rule based
RNAhybrid	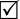	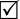		Rule based
microInspector	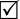	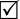		Rule based
MovingTargets	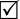	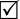		Rule based
Nucleus	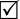	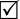		Rule based
Pictar	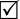	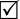	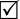	Data Driven: HMM
miTarget	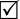	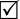		Data Driven: SVM
mirTarget	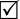	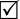	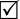	Data Driven: SVM
rna22	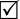	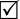		Data Driven: Markov Chain
SVMicro	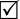	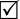	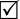	Data Driven: SVM
Targetboost				Data Driven: Boost
GenMiR++	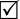	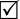	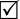	Data Driven: Bayesian Learning

**Table 4:** The Scoring Matrix Used by miRanda

	C	G	A	T	U	X
**C**	-3	+5	-3	-3	-3	-1
**G**	+5	-3	-3	+1	+1	-1
**A**	-3	-3	-3	+5	+5	-1
**T**	-3	+1	+5	-3	-3	-1
**U**	-3	+1	+5	-3	-3	-1
**X**	-1	-1	-1	-1	-1	-1
